# Mutational Analysis of Sse1 (Hsp110) Suggests an Integral Role for this Chaperone in Yeast Prion Propagation *In Vivo*

**DOI:** 10.1534/g3.113.007112

**Published:** 2013-08-01

**Authors:** Ciara Moran, Gemma K. Kinsella, Zai-Rong Zhang, Sarah Perrett, Gary W. Jones

**Affiliations:** * Yeast Genetics Laboratory, Department of Biology, National University of Ireland Maynooth, Maynooth, County Kildare, Ireland; † The Marie Curie Laboratory for Membrane Proteins, Department of Biology, National University of Ireland Maynooth, Maynooth, County Kildare, Ireland; ‡ National Laboratory of Biomacromolecules, Institute of Biophysics, Chinese Academy of Sciences, Chaoyang District, Beijing 100101, China

**Keywords:** *Saccharomyces cerevisiae*, prion, chaperone, Sse1, Hsp110, Hsp70, nucleotide exchange factor

## Abstract

The yeast Hsp110 chaperone Sse1 is a conserved protein that is a noncanonical member of the Hsp70 protein superfamily. Sse1 influences the cellular response to heat stress and has also been implicated in playing a role in the propagation of prions in yeast. Sse1 can seemingly exert its effects *in vivo* through direct or indirect actions by influencing the nucleotide exchange activity of canonical cytosolic Hsp70s. Using a genetic screen based on the inability to propagate the yeast [*PSI*^+^] prion, we have identified 13 new Sse1 mutants that are predicted to alter chaperone function through a variety of different mechanisms. Not only are these new Sse1 mutants altered in the ability to propagate and cure yeast prions but also to varying degrees in the ability to grow at elevated temperatures. The expression levels of chaperone proteins known to influence yeast prion propagation are unaltered in the Sse1 mutants, suggesting that the observed phenotypic effects are caused by direct functional alterations in these mutants. Mapping the location of the mutants onto the Sse1 crystal structure suggests that more than one functional alteration in Sse1 may result in changes in prion propagation and ability to function at elevated temperatures. All Sse1 mutants isolated provide essential functions in the cell under normal growth conditions, further demonstrating that essential chaperone functions *in vivo* can to some degree at least be detached from those related to propagation of prions. Our results suggest that Sse1 can influence prion propagation through a variety of different mechanisms.

Hsp110 proteins are a group of eukaryotic molecular chaperones that have been implicated in a variety of cellular functions. Several cytosolic Hsp110 protein variants have been described in eukaryotes, including HSPH1, Apg-1, Apg-2, and Grp170 in mammals (Vos *et al.* 2008; Kampinga *et al.* 2009). Hsp110 is represented in *Saccharomyces cerevisiae* by the Sse1 and Sse2 proteins. *SSE1* and *SSE2* constitute an essential gene pair in yeast (Trott *et al.* 2005) and although not essential in itself deletion of *SSE1* does confer a growth defect and stress-related phenotypes (Shirayama *et al.* 1993; Shaner *et al.* 2004, 2008). Sse1 was first isolated from yeast biochemically as a calmodulin-binding protein (Mukai *et al.* 1993) and genetically as a suppressor of a protein kinase A (PKA) mutant (Shirayama *et al.* 1993). Sse1 and Sse2 share a high degree of sequence identity (~76%) and are noncanonical members of the Hsp70 superfamily (Mukai *et al.* 1993). *SSE1* is expressed at moderately high levels under normal growth conditions and is further induced upon heat shock whereas *SSE2* transcripts are nearly undetectable at basal temperatures but are increased more than 20-fold upon heat shock (Mukai *et al.* 1993; Shirayama *et al.* 1993).

The Sse1 protein has been crystallized and established to be modular, built-up from Hsp70-like subdomains (Liu and Hendrickson 2007). Although Sse1 and canonical Hsp70 have diverged in function, certain structural features in Hsp70 have been conserved in Sse1. Mutational analysis revealed that particular mutant variants of Sse1 and Ssa1 (one of the major yeast cytosolic Hsp70s) result in similar phenotypic defects, supporting the hypothesis that Sse1 is an evolutionary vestige of Hsp70 (Liu and Hendrickson 2007). It has been reported that Sse1, like Ssa1, can recognize and bind hydrophobic peptide sequences with high affinity (Goeckeler *et al.* 2008) and can exhibit ATPase activity (Raviol *et al.* 2006a,b). However, the functional similarities end there, as Sse1 cannot functionally refold denatured proteins but instead acts as a “holdase” by binding denatured proteins and preventing their aggregation (Oh *et al.* 1999). This “holdase” function may serve a function in the peptide-refolding pathway carried out by other chaperones. Various Hsp110 homologs have been shown to accelerate the refolding of luciferase by Hsp70/Hsp40 machinery (Goeckeler *et al.* 2002).

Although specific intracellular functions of Hsp110 proteins are poorly characterized in comparison with various canonical Hsp70s, it has been suggested recently that they may act as the principal nucleotide exchange factor (NEF) for Hsp70. Sse1 was shown to act as a potent NEF for yeast cytosolic Hsp70 proteins Ssa1 and Ssa2 (Dragovic *et al.* 2006; Raviol *et al.* 2006b). This discovery followed soon after the discovery that Hsp110 proteins physically and functionally interact with their Hsp70-Ssa counterparts (Yamagishi *et al.* 2004; Shaner *et al.* 2005; Yam *et al.* 2005). Prior to these findings Fes1 was the only identified NEF for Ssa1 (Kabani *et al.* 2002a). The regulation of substrate binding by ATP hydrolysis and subsequent nucleotide exchange is a key component in maintaining correct *in vivo* function for all Hsp70 chaperones.

The general domain organization of Sse1 does reflect that of canonical Hsp70s. It consists of a N-terminal nucleotide-binding domain (NBD), a β-sandwich domain (SBD-β) and a three helical bundle domain (3HBD or SBD-α) toward the C-terminus. The Sse1 protein has a compact structure with tight interactions between the NBD and substrate-binding domain (SBD). Unlike Hsp70, the Sse1 SBD-α does not form a lid over its binding pocket but instead interacts with the flank of the Sse1 NBD (Polier *et al.* 2008). Sse1 is larger than Hsp70 as the result of insertions within the SBD and a C-terminal extension (Easton *et al.* 2000; Liu and Hendrickson 2007). Sse1 shares ~30% sequence identity with Ssa1 (Shaner *et al.* 2005; Yam *et al.* 2005). Like other Hsp70-Hsp110 interacting components, the sequence similarity between Sse1 and Ssa1 is largely confined to the NBD (Goeckeler *et al.* 2008).

Sse1 preferentially associates with Ssa1 *in vivo* (Shaner *et al.* 2005). The Hsp70 NBD is embraced by the NBD and SBD-α of Sse1, leading to the opening of the Hsp70 nucleotide-binding cleft. The Sse1 β-sandwich domain of the substrate binding cleft alternates away from the complex (Polier *et al.* 2010). It appears that almost the entire length of Sse1 is required for complex formation with Hsp70 (Shaner *et al.* 2004; Dragovic *et al.* 2006; Polier *et al.* 2008). Complex formation also requires Sse1 to be ATP-bound as this alters the NBD structure in a way that stabilizes it and allows it to bind Hsp70 (Shaner *et al.* 2006; Polier *et al.* 2008). Yeast Sse1 can also form a functional complex with human Hsp70, which reflects a high degree of conservation in the Hsp70-Hsp110 structure (Shaner *et al.* 2006).

The multidomain architecture of Sse1 suggests that it may play a role as a chaperone similar to Hsp70. However, the protein folding ability of canonical Hsp70s relies heavily on the conformational structural changes between the NBD and SBD upon ATP/ADP binding; such allostery appears absent in Sse1. The Sse1 substrate-binding pocket remains closed upon ATP binding, suggesting that any potential substrate-binding or chaperone activity inherent in Sse1 will be functionally distinct to Hsp70 (Andréasson *et al.* 2008).

Since the seminal paper by Wickner (1994), who proposed that the yeast non-Mendelian genetic elements [*PSI*^+^] and [*URE3*] are prions of the Sup35 and Ure2 proteins, respectively, the authors of many subsequent studies have shown this proposal to be correct and that a significant number of other fungal proteins have prion forming ability (Derkatch *et al.* 2001; Alberti *et al.* 2009). A variety of *in vitro* and *in vivo* studies have demonstrated an integral role for molecular chaperones in yeast prion propagation (reviewed in, Jones and Tuite 2005; True 2006; Perrett and Jones 2008; Masison *et al.* 2009). Most chaperone/prion studies have focused upon the yeast Hsp40/Hsp70/Hsp104 protein disaggregation machinery (Chernoff *et al.* 1995; Glover *et al.* 1997; Krzewska and Melki 2006; Shorter and Lindquist 2008), which has been shown to play an essential role in propagation of yeast prions. More recently, evidence has accumulated suggesting a role for yeast Hsp110 in prion formation and propagation. Studies have demonstrated Sse1 may be required for the *de novo* formation and propagation of [*PSI*^+^] (Fan *et al.* 2007; Kryndushkin and Wickner 2007; Sadlish *et al.* 2008). Current understanding suggests that Sse1 primarily influences prion formation and propagation due to its NEF function for Hsp70; however, Sse1 has been suggested to bind to early intermediates in Sup35 prion conversion and thus facilitate prion seed conversion independently of its NEF function (Sadlish *et al.* 2008). Overexpressed Sse1 was shown to increase the rate of *de novo* [*PSI*^+^] formation while deleting *SSE1* reduced [*PSI*^+^] prion formation; however, no effects on pre-existing [*PSI*^+^] were observed (Fan *et al.* 2007; Kryndushkin and Wickner 2007). In contrast, the overproduction or deletion of *SSE1* cured the [*URE3*] prion and mutant analysis suggests this activity is dependent on ATP binding and interaction with Hsp70 (Kryndushkin and Wickner 2007). Intriguingly, Sse1 has recently been shown to function as part of a protein disaggregation system that appears to be conserved in mammalian cells (Shorter 2011; Duennwald *et al.* 2012).

To gain further insight into the possible functional roles of Hsp110 in prion propagation, we have isolated an array of novel Sse1 mutations that differentially impair the ability to propagate [*PSI*^+^]. The locations of these mutants on the Sse1 protein structure suggest that impairment of prion propagation by Hsp110 can occur through a number of independent and distinct mechanisms. The data suggests that Sse1 can influence prion propagation not only indirectly through an Hsp70-dependent NEF activity, but also through a direct mechanism that may involve direct interaction between Sse1 and prion substrates.

## Materials and Methods

### Strains and plasmids

Strains and plasmids used and constructed in this study are listed and described in Table 1 and Table 2. Site-directed mutagenesis using the Quickchange kit (Stratagene) and appropriate primers were used to introduce desired mutations into plasmids. The G600 strain, the genome of which was recently sequenced (Fitzpatrick *et al.* 2011), was used to amplify *SSE* genes via polymerase chain reaction for cloning into pRS315. The human *HSPH1* gene (alternative name *HSP105*) was amplified from a cDNA clone purchased from Origene (Rockville, MD). All plasmids constructed in this study were verified by sequencing.

**Table 1:** Strains used in this study

Strain name	Genotype	Source
G600	*MAT***a** *ade2.1 SUQ5 kar1-1 his3 leu2 trp1 ura3* [*PSI^+^*]	Jones *et al.* (2004)
HLY100	G600 plus *Δsse1*	This study
HLY101	G600 mating type switched plus *Δsse2*	This study
HLY102	G600 isogenic +/*Δsse1* +/*Δsse2* [*PSI*^+^] diploid. Constructed by mating HLY100 and HLY101 and transforming with pRS316-SSE1	This study
CMY02	G600 plus *Δsse1 Δsse2* pRS316-SSE1, isolated as haploid segregant following sporulation of HLY102	This study
Y02146	*MAT****a*** *his3Δ1 leu2Δ0 met15Δ0 ura3Δ0 sse1*::*KanMX4*	Euroscarf
Y17167	*MAT****α*** *his3Δ1 leu2Δ0 lys2Δ0 ura3Δ0 sse2*::*KanMX4*	Euroscarf
CMY01	*MAT***a***/****α*** *his3Δ1/his3Δ1 leu2Δ0/leu2Δ0 lys2Δ0/+ +/met15Δ0 ura3Δ0 +/sse1*::*KanMX4 sse2*::*KanMX4/+* pRS316-SSE1 (constructed by mating Y02146 with Y17167 and transforming with pRS316-SSE1)	This study
CMY03	*MAT****α*** *his3Δ1 leu2Δ0 lys2D0 ura3Δ0 sse1*::*KanMX4 sse2*::*KanMX4* pRS316-SSE1 (haploid segregant following sporulation of CMY01)	This study
SB34	*MAT***a** *trp1-1 ade2-1 leu2-3,112 his3-11,15 ura2*::*HIS3 erg6*::*TRP1 dal5*::*ADE2* [*URE3*]	Bach *et al.* (2003)

**Table 2:** Plasmids used in this study

Plasmid Name	Description	Source
pRS315	Centromeric *Saccharomyces cerevisiae* shuttle vector, *LEU2* marker	(Sikorski and Hieter 1989)
pRS316	Centromeric *Saccharomyces cerevisiae* shuttle vector, *URA3* marker	(Sikorski and Hieter 1989)
pRS423	2μ *Saccharomyces cerevisiae* high copy plasmid, *HIS3* marker	(Christianson *et al.* 1992)
pC210	*SSA1* under control of *SSA2* promoter, *LEU2* marker	(Schwimmer and Masison 2002)
pRS315-SSE1	*SSE1* ± 500bp cloned into pRS315, *LEU2* marker	This study
pRS316-SSE1	*SSE1* ± 500bp cloned into pRS315, *URA3* marker	This study
pRS315-SSE2	*SSE2* ± 500bp cloned into pRS315, *LEU2* marker	This study
pRS315-SSE2^Q504E^	Site directed mutagenesis of pRS315-SSE2 to produce Q504E	This study
pRS315-SSE2^G616D^	Site directed mutagenesis of pRS315-SSE2 to produce G616D	This study
pRS315-SSE2^Q504E/G616D^	Site directed mutagenesis of pRS315-SSE2^Q504E^ to produce Q504E+G616D	This study
pRS423-FES1	*FES1* ±500bp cloned into pRS423, *HIS3* marker	(Jones *et al.* 2004)
pC-HSPH1	*HSPH1* under control of *SSA2* promoter, *LEU2* marker	This study
pRS423-CIA1	*CIA1* ± 500bp cloned into pRS423, *HIS3* marker	This study

### Media and genetic methods

Standard media was used throughout this study as previously described (Guthrie and Fink 1991). Monitoring of [*PSI*^+^] was carried out as described (Jones and Masison 2003). Briefly, the presence of [*PSI*^+^] (the non-functional aggregated form of Sup35) and *SUQ5* causes efficient translation read through of the *ochre* mutation in the *ade2-1* allele. Non-suppressed *ade2-1* mutants are Ade^-^ and are red when grown on medium containing limiting amounts of adenine due to the accumulation of a pigmented substrate of Ade2. Partial suppression of *ade2-1* by [*PSI*^+^] allows growth without adenine and eliminates the pigmentation (Cox 1965).

Monitoring of [*URE3*] again made use of the red/white selection based on the *ADE2* gene. The strain SB34 has *ADE2* under control of the *DAL5* promoter. In [*URE3*] cells expression of the *DAL5* promoter is high because of the action of Gln3. In [ure-0] cells soluble Ure2 can interact with Gln3 and prevent transcription from the *DAL5* promoter. Hence, when [*URE3*] is present the SB34 strain will grow on medium lacking adenine and is white on medium with limiting adenine. When [ure-0] this strain will not grow on medium lacking adenine and is red on medium with limiting adenine.

### Generation of *SSE1* mutant library

Plasmid pRS315-SSE1 was subjected to treatment with hydroxylamine for 60 min (Schatz *et al.* 1988). This treatment resulted in mutation frequencies of around 8% for this plasmid (G. W. Jones, unpublished data).

### Isolation of Sse1 mutants that impair [*PSI*^+^] prion propagation

Sse1 mutants were isolated using the plasmid shuffle technique. Strain CMY02 was transformed with the *SSE1* mutagenized plasmid library. Transformed cells were selected on medium lacking leucine. Any red or dark-pink colonies were scored at this point as potential dominant *SSE1* mutants that could weaken [*PSI*^+^]. Transformation plates were replica plated onto medium-containing limiting amounts of adenine and also 5-fluoro-orotic acid, a chemical that selects against *URA*^+^ cells and hence against the presence of the pRS316-SSE1 plasmid. Colonies appearing red or dark-pink at this stage were scored as potentially harboring a mutant *sse1* allele that cannot maintain [*PSI*^+^]. All potential *sse1* mutant containing plasmids were isolated and retransformed back into CMY02 and analyzed for their effects upon [*PSI*^+^]. After retransformation, the color phenotype of colonies was scored subjectively from 0 to 9, with 0 being white and 9 being red (Loovers *et al.* 2007).

### Assaying mutant effects on [*URE3*]

Effects on [*URE3*] were assayed as previously described (Loovers *et al.* 2007). To summarize, SB34 was grown to log phase growth under conditions that maintain [*URE3*] (medium lacking adenine). Cells were transformed with wild-type (WT) or mutant *SSE1* alleles and transformants were selected on medium lacking leucine. At this stage all cells (at least 100) were scored for color phenotype on the basis of being white, red or sectored.

### Mapping mutants onto crystal structure of Sse1 and molecular modeling

Structures for Sse1 (2QXL; (Liu and Hendrickson 2007) and for Sse1 in complex with Ssa1 (3D2F; (Polier *et al.* 2008) were obtained from the Protein Data Bank. Molecular modeling to complete gap regions, introduce point mutations (100 models each), and for visualization was carried out using Molecular Operating Environment, version 2009.10 (Chemical Computing Group Inc., 2009). Images were generated using pyMol (DeLano 2002).

### Western analysis

Western analysis was performed essentially as described previously (Jones and Masison 2003). Hsp70 monoclonal antibody was purchased from Cambridge Bioscience (SPA822), Sse1 polyclonal antibody was a gift from Jeff Brodsky (University of Pittsburgh), and Hsp104 polyclonal antibody was a gift from John Glover (University of Toronto).

## Results

### Isolation of novel mutants of *SSE1* that impair [*PSI*^+^] prion propagation

**Figure 1:**
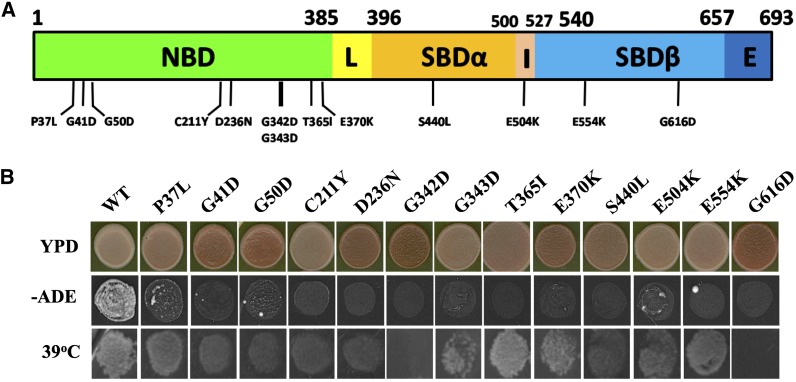
(A) Sse1 mutants that impair prion propagation are located in various domains of the protein. Numbers above refer to amino acids that define the boundaries of the nucleotide-binding domain (NDB), linker region (L), substrate-binding domain (SBD), Hsp110 insertion region (I), and Hsp110 extension region (E). Mutants isolated that impair prion propagation are indicated below the linear structure. (B) Phenotype of Sse1 mutants that impair prion propagation. Top panel shows color on YPD, middle panel depicts growth on medium lacking adenine, and bottom panel is growth on YPD at 39°.

Applying the plasmid shuffle technique as described in *Materials and Methods* we have identified 13 new mutants of Sse1 that impair propagation of the [*PSI*^+^] prion (Figure 1, Table 3). Nine of these mutants are located in the NBD and like previous studies highlight the general functional importance of correct ATPase regulation of Hsp70 chaperones in yeast prion propagation (Jones and Masison 2003; Loovers *et al.* 2007). The mutants had a wide range of effects on propagation of [*PSI*^+^], with some being unable to propagate the prion at all (G41D, G50D, D236N, G342D, E370K, and G616D) to others having minor effects on color phenotype (P37L, C211Y; Table 3 and Figure 1B). The presence or absence of [*PSI*^+^] in all mutants was confirmed by mating with a [*psi*^−^] strain followed by sporulation of any [*PSI*^+^] diploids to confirm non-Mendelian segregation and subsequent growth on guanidine hydrochloride to cure the prion (data not shown). As expected, all Sse1 mutants that could not propagate [*PSI*^+^] could not grow on medium lacking adenine (Figure 1B). However, surprisingly, all other Sse1 mutants, even ones that had an apparently mild affect on [*PSI*^+^], also grew very poorly or not at all on medium lacking adenine (Figure 1B). The reason for these growth results is unknown but perhaps suggests Sse1 may be involved in cellular metabolic pathways that can result in complex nutritional phenotypes. Significantly, none of the mutants had a major adverse effect on cell growth at 30°, suggesting that each mutant is capable of carrying out the essential cellular functions of Sse1 (Table 3). However, at 39° there are major differences in the abilities of the mutants to grow (Table 3, Figure 1B). Deletion of *SSE1* causes a 39° temperature-sensitive phenotype (Shaner *et al.* 2008) and therefore it appears that a subset of mutants (G50D, G342D, S440L, G616D) are effectively nonfunctional at this elevated temperature. Other mutants appear to provide either WT levels of activity (P37L, T365I, E554K) or some intermediate or reduced level of Sse1 functionality (G41D, C211Y, D236N, G343D, E370K, E504K).

**Table 3:** Relative effects of *SSE1* mutants on [*PSI*^+^] prion propagation and cell growth

Sse1 Mutation	Times Isolated*^a^*	Color Pre-5-FOA*^b^*	Color post-5-FOA*^b^*	Growth at 39°*^c^*	Generation time (% of WT)*^d^*
None	−	0	0	+++++	100
P37L	1	2	3	+++++	96
G41D	3	3	8	++	100
G50D	3	4	8	+	101
C211Y	1	3	2	++	93
D236N	1	4	9	++	110
G342D	3	3	9	−	114
G343D	1	3	4	+++	104
T365I	1	3	5	+++++	104
E370K	1	2	9	+++	107
S440L	1	2	6	+	97
E504K	1	2	4	+++	118
E554K	2	3	4	+++++	101
G616D	1	2	9	−	113

5-FOA, 5-fluoro-orotic acid; WT, wild type.

a Number of independent times isolated in the mutant screen.

b Color: 0, white [*PSI*^+^]; nine, Red, [*psi*^-^]; FOA, selection against presence of WT *SSA1 URA3* plasmid.

c Relative growth after 2 d at 39°.

d Doubling time in minutes expressed as a % of CMY02 harboring WT *SSE1*.

### Effects of *FES1* overexpression on the ability of Sse1 mutants to propagate [*PSI*^+^]

**Figure 2:**
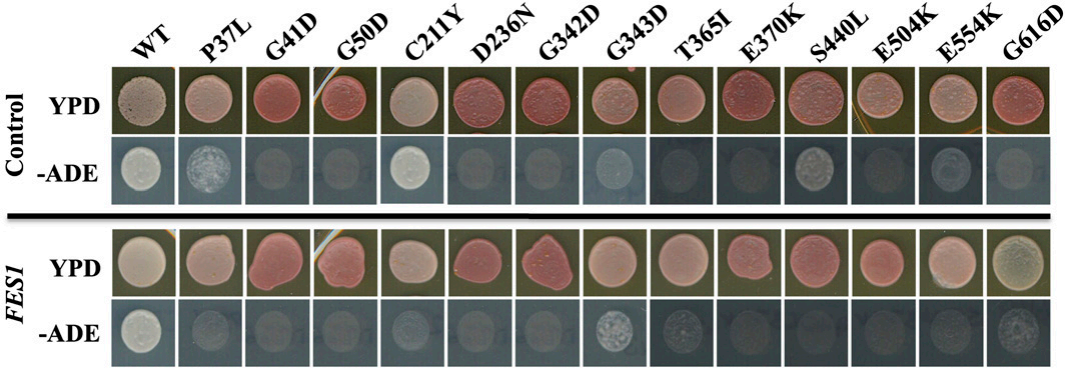
Sse1 mutants exhibit a complex growth phenotype when grown on medium lacking adenine. The absence of histidine and the presence of *FES1* can affect the ability of Sse1 mutants to grow on medium lacking adenine. Top section is growth in presence of either vector control or overexpression of *CIA1*, and bottom section is in the presence of over-expressed *FES1*. The results shown are representative of three independent experiments, for controls this constitutes two experiments with vector only and one with *CIA1* overexpression.

Both Fes1 and Sse1 have been shown to be NEFs for cytosolic Hsp70s (Kabani *et al.* 2002b; Dragovic *et al.* 2006; Raviol *et al.* 2006b) We therefore assessed the ability of Fes1 to complement the prion propagation defect of this novel set of Sse1 mutants. To do this we carried out plasmid shuffle analysis for each Sse1 mutant in the presence of over-expressed Fes1 (Figure 2). As a negative control plasmid shuffle analysis was also carried out in the presence of either pRS423 (vector only) or pRS423 harboring the *CIA1* gene ±500 bp. *CIA1* is a yeast gene that has not been implicated in altering yeast prion propagation. After growth on 5-fluoro-orotic acid media also lacking histidine (to maintain selection for pRS423 based plasmids), cells were placed onto YPD to assess color and –ADE –HIS medium to assess the ability to grow on medium lacking adenine. Although the color phenotype on YPD for Sse1 WT or mutant cells harboring the vector or overexpressing *FES1* is consistent with presence of Sse1 alone (compare Figure 1B YPD panel with Figure 2 control and *FES1* YPD panels), the ability of some CMY02 Sse1 mutant cells to grow on medium lacking adenine is influenced greatly by the absence of histidine (compare Figure 1B –ADE panel with Figure 2 control and *FES1* –ADE panels). Only G616D appears altered in color on YPD by the presence of *FES1* overexpression. However, this color change does not correlate with a significant increased ability to grow on –ADE medium (Figure 2). Comparing the effects of vector only to overexpressed *FES1*, a clear difference in ability to grow on –ADE medium is observed for some mutants; P37L, C211Y, S440L, and E554K grow less well on –ADE in the presence of overexpressed *FES1*, whereas G343D and T365I grow slightly better in the presence of overexpressed *FES1* (Figure 2), suggests that increases in Hsp70 (Ssa) NEF activity are able to influence some phenotypes of this subset of Sse1 mutants. Currently, we have no explanation for the complex but reproducible –ADE phenotype of these novel Sse1 mutants shown in Figures 1B and 2.

### Sse1 mutants are defective in ability to cure [*URE3*] prion

A previous study has highlighted the ability of overexpressed Sse1 to impair propagation of the yeast prion [*URE3*] (Kryndushkin and Wickner 2007). Similarly we found that in the SB34 strain background (Bach *et al.* 2003) the introduction of an extra copy of *SSE1* under control of its native promoter was capable of causing a significant impairment of [*URE3*] (Table 4). We therefore assessed the ability of the Sse1 mutants to impair [*URE3*] propagation using this assay. In contrast to WT Sse1 and in contrast to the diverse phenotypic effects observed in [*PSI*^+^] prion propagation and temperature sensitivity assays, we found that all thirteen novel Sse1 mutants were unable to significantly impair [*URE3*] propagation in the SB34 strain (Table 4). This suggests either a common functional change or defect within these mutants with respect to the ability to cure [*URE3*] or that more than one functional alteration in Sse1 can impair [*URE3*] curing ability.

**Table 4:** Relative effects of Sse1 mutants on ability to cure [*URE3*]

Sse1 Mutation	% White	% Red	% Sectored
None/WT	48	13	39
P37L	90	3	7
G41D	96	1	3
G50D	94	4	2
C211Y	92	4	5
D236N	98	1	1
G342D	95	2	3
G343D	84	7	9
T365I	84	11	5
E370K	94	2	4
S440L	87	5	8
E504K	87	4	9
E554K	86	4	10
G616D	83	4	13
Vector only	96	2	2

Colony color was scored subjectively as for Table 1. Colony percentage is given after transformation of *SSE1* mutant into SB34 as described in *Materials and Methods*. WT, wild type.

### Chaperone abundance in Sse1 mutants

**Figure 3:**
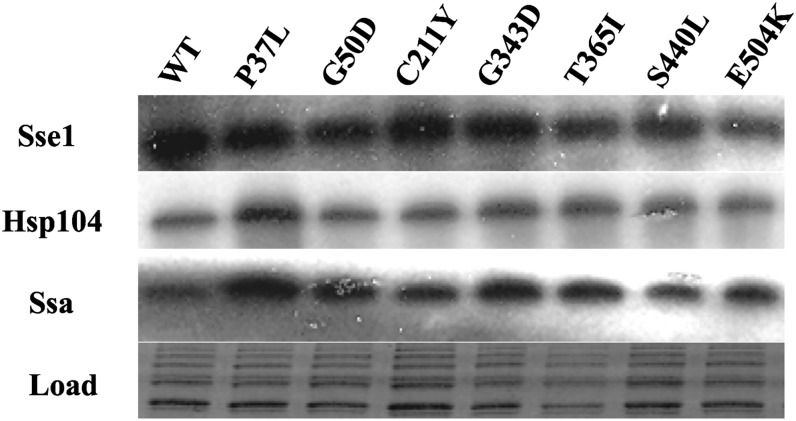
No change in protein levels of chaperones known to alter [*PSI*^+^] propagation in Sse1 mutants. Western blot analysis to measure protein levels of Sse1, Hsp70 (Ssa), and Hsp104. After initial blotting with anti-Sse1 antisera, the membrane was stripped and subsequently probed with Hsp104 and Hsp70 antibodies. The membrane was stained with Amido Black to show loading.

It is well documented that certain chaperones play an essential role in prion maintenance and alteration in expression levels can affect [*PSI*^+^] propagation (for review see (Jones and Tuite 2005)). We therefore measured Sse1, Hsp104 and the Hsp70 (Ssa) chaperone family expression levels in all the Sse1 mutants. Figure 3 (and data not shown) shows that no major differences in chaperone expression levels exist between any mutants compared to wild-type Sse1. Only the P37L mutant appeared to have slightly increased levels of Hsp104 and Ssa, but taking into account previous findings these are unlikely to be the cause of any prion or temperature-related phenotypes (Jung *et al.* 2000; Jones and Masison 2003; Loovers *et al.* 2007). In addition we also measured levels of Hsp70 co-chaperones Ydj1 and Sis1 and found similar amounts of these Hsp40s within the Sse1 mutants analyzed in Figure 3 compared to wild type (data not shown). Therefore, the phenotypic changes in prion propagation and growth at high temperatures observed in these novel Sse1 mutants is most likely not due to indirect changes in chaperone expression levels.

As shown in Figure 1, a number of Sse1 mutants are unable to grow at 39°. One possible explanation for this phenotype is that such Sse1 mutants are unstable at this temperature. We therefore used Western blotting to assess the stability of Sse1 mutants following exposure to 39° for 1 hr and found no difference in stability between any Sse1 mutants compared to wild-type protein (data not shown).

### Location of mutants on crystal structure of Sse1: functional implications

**Figure 4:**
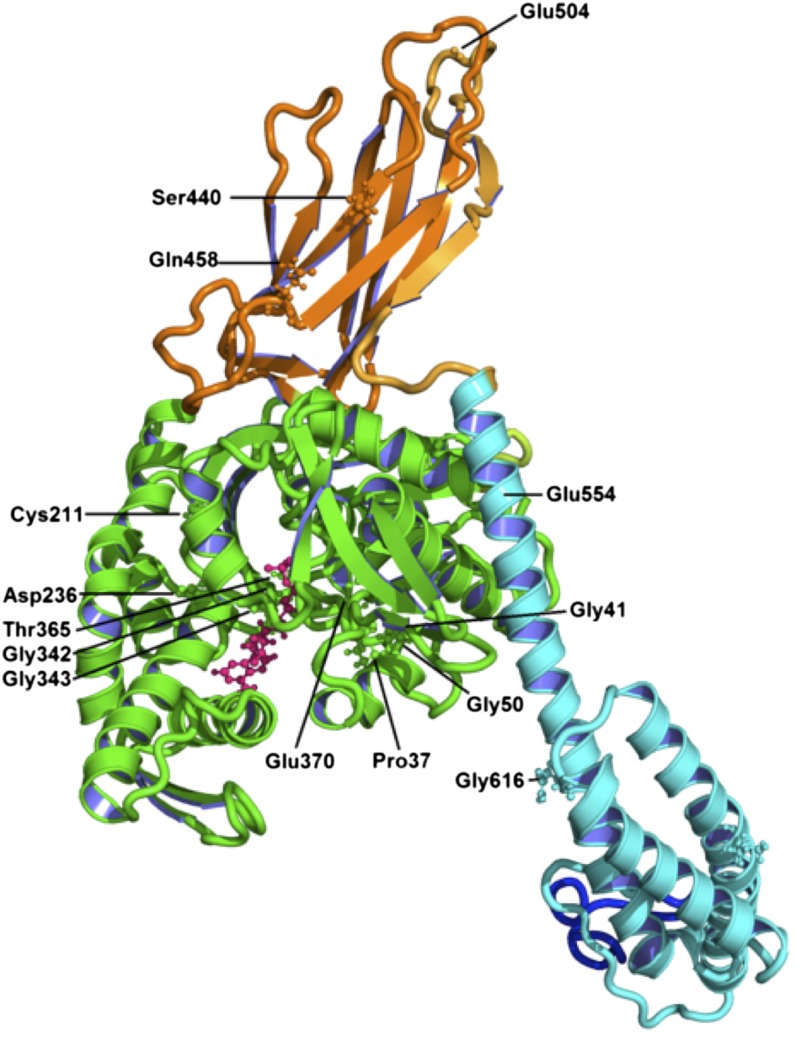
Mapping of mutations onto Sse1 structure. (A) Structural model of Sse1 (PDB: 2QXL) with the residues of interest highlighted and in ball and stick format. Domains are colored to correspond to Figure 1A. Images were generated using Pymol (DeLano 2002).

The crystal structure of the Sse1 protein alone and in complex with cytosolic Hsp70 has been determined (Liu and Hendrickson 2007; Polier *et al.* 2008; Schuermann *et al.* 2008). To gain insight into possible functional consequences of this new set of Sse1 mutations we mapped mutated residues onto available Sse1 structures and used molecular modeling to predict possible localized structural changes and functional implications (Figure 4, Table 5 and Supporting Information, File S1). Of the nine mutants identified within the NBD four are predicted to affect ATP binding (P37L, G342D, G343D, E370K), three to alter interaction with cytosolic Hsp70 (G41D, T365I, E370K), and three remain unclear (G50D, C211Y, D236N) (Table 5, File S1). The four mutants isolated in the SBD domain are predicted to alter either Sse1 interaction with cytosolic Hsp70 (E554K, G616D, see Figure S3), substrate binding (S440L), or protein−protein interactions (E504K) (Table 5 and Supplemental Information).

**Table 5:** Predicted structural effects of mutants

Mutation	Location	Predicted Effect
P37L	β-sheet within NBD	ATP binding
G41D	β-sheet within NBD	Hsp70 interaction
G50D	α-helix within NBD	Unclear
C211Y	β-sheet within NBD	Unclear
D236N	α-helix within NBD	Unclear
G342D	ATP binding pocket of NBD	ATP binding
G343D	ATP binding pocket of NBD	ATP binding
T365I	Loop region within NBD	Hsp70 interaction
E370K	α-helix within NBD	ATP binding/Hsp70 interaction
S440L	α-helix within SBDβ	Substrate binding
E504K	Within insertion region of SBDβ	Protein-protein interactions
E554K	α-helix within SBDα	Protein-protein interactions
G616D	Loop region within SBDα	Hsp70 interaction

NBD, nucleotide-binding domain; SBD, substrate binding domain.

### Sse2 and [*PSI*^+^] propagation

**Figure 5:**
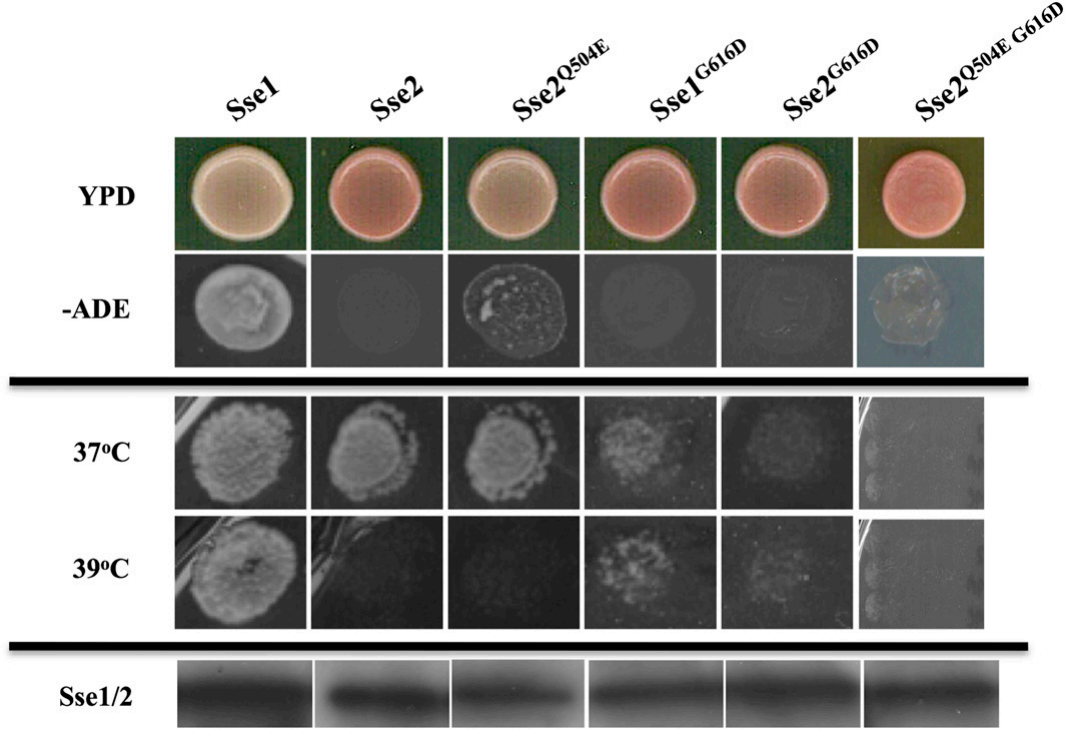
Phenotypic analysis of yeast cells expressing Sse2 as the sole source of Hsp110. Growth of Sse1, Sse2, and Sse2 derived mutants on medium lacking adenine (top growth panels) and at elevated temperature (lower growth panels). Western blotting was used to assess expression levels of Sse1, Sse2, and mutants (bottom panels).

Figure S1 shows an alignment of Sse1 and Sse2. Although these proteins share 76% identity, Sse2 is unable to compensate for Sse1 in terms of [*PSI*^+^] prion propagation or growth at higher temperatures (Figure 5; Sadlish *et al.* 2008; Shaner *et al.* 2008). All but one of our novel Sse1 mutated residues is conserved in Sse2, the nonconserved residue corresponding to position E504 in Sse1, which is Q504 in Sse2. We reasoned that the inability of Sse2 to propagate [*PSI*^+^] could be influenced by this residue difference. Using site-directed mutagenesis, we created a Q504E mutant version of Sse2 and assessed the ability of this protein to propagate [*PSI*^+^]. In contrast to wild-type Sse2, Sse2^Q504E^ is able to propagate [*PSI*^+^], although not to the same degree as Sse1 (Figure 5). Interestingly, although [*PSI*^+^] propagation is restored to some degree in Sse2^Q504E^, the ability to grow at 39° is not (Figure 5). In addition to rendering Sse1 unable to propagate [*PSI*^+^], the G616D mutation was one of two Sse1 mutants that also caused a 37° temperature-sensitive phenotype (Figure 5 and data not shown). Similarly, when G616D is introduced into Sse2 the same phenotype was observed, indicating conservation of functional importance of this residue in these two proteins. Combining Q504E and G616D in the Sse2 protein produces similar phenotypes as observed for Sse1 (Figure 5) and further demonstrates the functional conservation between these residues within yeast Sse proteins.

### Functional complementation of an *sse1 sse2* double deletion strain by *FES1* and human *HSPH1* is dependent on strain background

**Figure 6:**
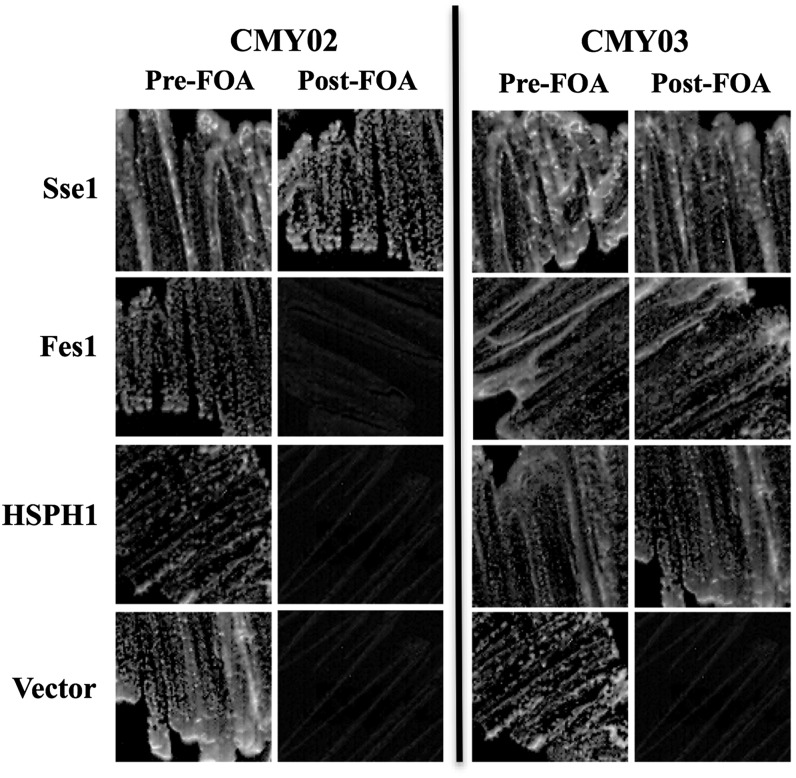
Complementation of *sse1 sse2* deletion strain by overexpression of *FES1* or mammalian *HSPH1*. Growth of *sse1 sse2* expressing *FES1* or *HSPH1* in place of *SSE1* was assessed in two strain backgrounds; CMY02 (G600 background, left section) and CMY03 (BY background, right section). As expected, vector only control produced no growth in either background.

A previous study has reported that the essential and prion-related functions of Sse1 were mainly related to the ability of the protein to function as a NEF for Hsp70. This was demonstrated by the ability of Fes1 and a N-terminally truncated Snl1 protein to complement the lethality of an *sse1sse2* double deletion strain (Sadlish *et al.* 2008). We therefore assessed whether Fes1 and the closest human Sse1 ortholog HSPH1 (Figure S2) could propagate [*PSI*^+^] in the G600 background. We found that both Fes1 and HSPH1 were unable to complement essential Sse1/2 functions in the CMY02 strain (Figure 6), and hence we were unable to assess whether [*PSI*^+^] could be propagated. The inability of Fes1 and HSPH1 to functionally substitute for deletion of *sse1* and *sse2* is strain specific as both were able to provide essential Sse1/2 functions in strain CMY03, which was constructed in the BY4741 background (Figure 6, Table 1). The cause of this difference in strain complementation is as yet unknown.

## Discussion

We have identified 13 novel mutations in Sse1 that have varying effects on both the ability of *S. cerevisiae* to propagate the [*PSI*^+^] prion and also to grow at increased temperatures. In contrast, all Sse1 mutants were similarly impaired in the ability to cure the [*URE3*] prion following overexpression. The phenotypic effects of these mutants appear to result from functional changes in the Sse1 protein and are not due to changes in expression levels of other chaperones known to influence prion propagation. Given the varied locations of these mutants in the Sse1 molecule and their predicted structural effects, we provide evidence to suggest that Sse1 can influence both yeast prion propagation and heat shock response in a variety of ways, which are potentially direct or indirect in manner.

Recently, Sse1 has been shown to play a role in the disaggregation of amyloid aggregates, including Sup35 (Shorter 2011; Rampelt *et al.* 2012). In combination with Hsp40 and Hsp70, Sse1 can dissolve amyloid aggregates albeit at a slower rate than Hsp104. Sse1 also can enhance disaggregation by Hsp104 (in the presence of Hsp40 and 70). This new role for Hsp110 proteins is conserved across species and provides the first clearly identified protein disaggregation machinery in mammalian cells (Shorter 2011; Duennwald *et al.* 2012). This newly discovered biochemical activity of Sse1 and the fact that Sse1 appears to interact directly with Sup35 prions *in vivo* (Bagriantsev *et al.* 2008) suggests that this chaperone may play a more direct and active role in modulating the propagation of yeast prions than was previously thought. Sse1 may influence prion propagation through influencing Ssa1 function but may also do so through interacting directly with prion aggregates. The diverse range of Sse1 mutants we have isolated in this genetic screen and their potential functional implications (Table 5 and Supplemental Information), supports this proposal.

Phenotypic analysis of the Sse1 mutants revealed subsets of mutants that were impaired to varying degrees in their ability to grow at elevated temperatures (Figure 1, Table 3). These results were very clear-cut and presumably are a consequence of altered Sse1 function due to the structural alterations. However, [*PSI*^+^] and corresponding adenine growth phenotypes of the mutants was very complex (Figure 1 and Figure 2, Table 3). The colony color phenotype initially used for screening and assessing the presence of [*PSI*^+^] was very clear; that is to say, the presence or absence of [*PSI*^+^] correlated well with the colony color phenotype. In contrast, the ability to grow on medium lacking adenine did not correlate well for all the mutants. As expected those mutants shown not to propagate [*PSI*^+^] did not grow on –ADE medium. However, some Sse1 mutants confirmed as maintaining [*PSI*^+^] were also unable to grow on medium lacking adenine. Furthermore, the removal of histidine from the medium can influence the ability of some Sse1 mutants to grow in the absence of adenine and the subsequent overexpression of *FES1* can further affect this phenotype (Figure 2). Currently, we do not have any explanation for this very complex but reproducible phenotype, but speculate that Sse1 may play a role (direct or indirect) in modulating the histidine and/or adenine biosynthetic pathways. Both pathways are part of the “super-pathway of histidine, purine and pyrimidine biosynthesis” (*Saccharomyces* Genome Database) and converge on production of the biosynthetic intermediate aminoimidazole carboxamide ribonucleotide, accumulation of which can be toxic to the cell. If Sse1 is involved in modulating this super-pathway then our mutants may be affected in the ability to synthesize either histidine or adenine (or both) and toxic intermediates on this pathway may also be caused to accumulate. The addition of histidine or adenine to growth medium would have the effect of switching off these pathways and therefore suppressing any impaired growth phenotype due to the accumulation of toxic intermediates.

Given the variation in the effects of mutants upon [*PSI*^+^] propagation and also heat shock we were surprised to discover that all the Sse1 mutants were unable to efficiently cure the [*URE3*] prion. In a previous study, Kryndushkin and Wickner (2007) demonstrated that overexpression of the Sse1^G223D^ mutant (reduction in Sse1 ATPase, interaction with Ssa1 and loss of Ssa1 NEF activity) was unable to cure [*URE3*] whereas Sse1^K69M^ (can bind ATP but defective in hydrolysis) efficiently cured [*URE3*]. Thus, it seemed that efficient Sse1 NEF activity is required to cure [*URE3*]. Our data suggest that this may be an oversimplification. The clear phenotypic differences observed for the Sse1 mutants in respect of [*PSI*^+^] propagation and heat shock cannot be explained by a single unifying change in Sse1 function in all mutants. This suggestion is also supported by the location of the mutations on the Sse1 structure. Therefore it appears that a variety of mechanisms that alter Sse1 function can alter the ability to cure [*URE3*]. However, it should be noted that the ability to cure [*URE3*] could be influenced by the prion variant that is present in the cells. The [*URE3*] variants present in the SB34 strain and strains used by Kryndushkin and Wickner (2007) have not been compared directly.

Although Sse1 and Sse2 share a high degree of amino acid sequence identity (Figure S1), Sse2 is unable to compensate fully for the loss of Sse1. Sse2 has previously been shown to compensate for all *sse1*-deficient phenotypes at 30° (Shaner *et al.* 2004); however, this is not the case for [*PSI*^+^] propagation (Figure 5). In the G600 strain background, the loss of Sse1 function causes loss of [*PSI*^+^], demonstrating a clear distinction in the activities of Sse1 and Sse2 at 30°. The fact that the Sse1 mutants that have the greatest impairment of [*PSI*^+^] propagation are predicted to be altered in ATP binding and interaction with Hsp70 suggests that *in vivo* these activities are where Sse1 and Sse2 will differ the most. However, of all 13 mutated residues isolated in Sse1 identified as altering prion propagation, only one (E504) is not conserved in Sse2 (Q504) (Figure S1). We reasoned that this residue contributes to the inability of Sse2 to propagate [*PSI*^+^]. When this residue is mutated to create Sse2^Q504E^ [*PSI*^+^] can be propagated albeit not to the same extent as Sse1 (Figure 5). This result suggests that this residue is a key factor in dictating divergence of Sse1 and Sse2 function, and this residue is not predicted to alter ATP-binding or interaction with Hsp70. Hence, it appears that the *in vivo* differences in function between Sse1 and Sse2 are probably attributable to a number of different modifications in activity and not solely to one distinct difference. Clearly the interaction with Hsp70 is a key factor for *in vivo* function of Sse1 and Sse2 as demonstrated by the conserved effects of the G616D mutation (Figure 5). The combining of the Q504E and G616D mutation in the Sse2 protein produces similar phenotypic responses as for the same Sse1 variant. This indicates the functional conservation of these residues in yeast Sse proteins.

The conservation of essential *in vivo* functions carried out by Sse1 is clearly shown by the ability of the closest human homolog HSPH1 to complement the growth phenotype of a *sse1sse2* deletion strain. A recently characterized Hsp110 ortholog from *Arabidopsis thaliana* (AtHsp70-15) was shown to be unable to complement heat shock phenotypes of a *sse1* deletion strain constructed in the W303 background (Jungkunz *et al.* 2011). The G600 background used in this study is currently the most closely related sequenced laboratory strain to the original reference yeast strain S288C (Fitzpatrick *et al.* 2011) and yet there is a background-specific effect on the ability of HSPH1 to complement Sse defects. Hence, testing the AtHsp70-15 cDNA for complementation of *sse* deletion strains in different yeast backgrounds is certainly worth investigating and may demonstrate further the conservation of Hsp110 essential functions across diverse species.

The isolation of a set of new Sse1 mutants that alter yeast prion propagation has provided further evidence of an integral role for this chaperone in modulating the propagation of [*PSI*^+^] and perhaps the growing list of confirmed yeast prions. This set of newly characterized Sse1 mutants provides the opportunity for detailed biochemical assessment to address the causes of subtle differences that may exist in the functional alterations of Sse1 that effect activities in prion propagation as compared to other roles in heat shock or stress resistance. The canonical Hsp70 (Ssa) family is well characterized in its ability to modulate prion propagation and how this function can be distinct from roles in the heat shock response (Jung *et al.* 2000; Jones and Masison 2003; Loovers *et al.* 2007). To some degree, the same may be true for Sse1.

## Supplementary Material

Supporting Information
