# Supplementary material for: Mutational Analysis of Sse1 (Hsp110) Suggests an Integral Role for this Chaperone in Yeast Prion Propagation In Vivo
Source: G3 (Bethesda). 2013 Aug 1;3(8):1409–18. doi: 10.1534/g3.113.007112 (PMC3737180; doi:10.1534/g3.113.007112)
Supplement: Supporting Information [file supp_3_8_1409__index.html]

Mutational Analysis of Sse1 (Hsp110) Suggests an Integral Role for this Chaperone in Yeast Prion Propagation In Vivo — Supporting Information 

# Mutational Analysis of Sse1 (Hsp110) Suggests an Integral Role for this Chaperone in Yeast Prion Propagation *In Vivo*

## Supporting Information for Moran *et al.*, 2013

**Files in this Data Supplement:**

- Supporting Information - Figures S1-S3 and File S1 (PDF, 589 KB)
- Figure S1 - Sse1p (top sequence) and Sse2p alignment (PDF, 104 KB)
- Figure S2 - Alignment of Sse1p with HSPH1 (PDF, 106 KB)
- Figure S3 - Interaction of Hsp110 with Hsp70 (PDF, 515 KB)
- File S1 - Possible Sse1 mutant effects in more detail (PDF, 106 KB)
