# Supplementary material for: Mutational Analysis of Sse1 (Hsp110) Suggests an Integral Role for this Chaperone in Yeast Prion Propagation In Vivo
Source: G3 (Bethesda). 2013 Aug 1;3(8):1409–18. doi: 10.1534/g3.113.007112 (PMC3737180; doi:10.1534/g3.113.007112)
Supplement: Supporting Information [file supp_g3.113.007112_007112SI.pdf]

G3·Genes | Genomes | Genetics

Mutational Analysis of Sse1 (Hsp110) Suggests an Integral Role for this Chaperone in Yeast Prion Propagation *In Vivo*

Ciara Moran[a], Gemma K. Kinsella[b], Zai-Rong Zhang[c,d], Sarah Perrett[c], and Gary W. Jones[a]

[a]Yeast Genetics Laboratory, Department of Biology, National University of Ireland Maynooth, Maynooth, County Kildare, Ireland.
[b]The Marie Curie Laboratory for Membrane Proteins, Department of Biology, National University of Ireland Maynooth, Maynooth, County Kildare, Ireland.
[c]National Laboratory of Biomacromolecules, Institute of Biophysics, Chinese Academy of Sciences, 15 Datun Road, Chaoyang District, Beijing 100101, China.

[d]*Current address:* Department of Cell Biology, Nanobiology Institute, Yale School of Medicine, 850 West Campus Drive, Orange, CT 06516, USA

DOI: 10.1534/g3.113.007112

```
  1 MSTPFGLDLGNNNSVLAVARNRGIDIVVNEVSNRSTPSVVGFGPKNRYLG  50
    |||||||||||||||||||||||||:||||||||||||:|||||:|||||
  1 MSTPFGLDLGNNNSVLAVARNRGIDVVVNEVSNRSTPSLVGFGPRNRYLG  50

 51 ETGKNKQTSNIKNTVANLKRIIGLDYHHPDFEQESKHFTSKLVELDDKKT 100
    |:||.|||||:||||.||||||||.:..|:|:.|:|.||||||:|.:.|.
 51 ESGKTKQTSNVKNTVENLKRIIGLKFKDPEFDIENKFFTSKLVQLKNGKV 100

101 GAEVRFAGEKHVFSATQLAAMFIDKVKDTVKQDTKANITDVCIAVPPWYT 150
    |.||.|.|:.||||||||.||||||||.||:::||::|||||:|||.||:
101 GVEVEFGGKTHVFSATQLTAMFIDKVKHTVQEETKSSITDVCLAVPVWYS 150

151 EEQRYNIADAARIAGLNPVRIVNDVTAAGVSYGIFKTDLPEGEEKPRIVA 200
    ||||||||||||||||||||||||||||.||||:||.|||..||||||:.
151 EEQRYNIADAARIAGLNPVRIVNDVTAAAVSYGVFKNDLPGPEEKPRIIG 200

201 FVDIGHSSYTCSIMAFKKGQLKVLGTACDKHFGGRDFDLAITEHFADEFK 250
    .||||||:||||||||:||::||||||.||||||||||.||||||||:||
201 LVDIGHSTYTCSIMAFRKGEMKVLGTAYDKHFGGRDFDRAITEHFADQFK 250

251 TKYKIDIRENPKAYNRILTAAEKLKKVLSANTNAPFSVESVMNDVDVSSQ 300
    .|||||||:|||||||||.|||||||||||||.|||||||||:|:|||||
251 DKYKIDIRKNPKAYNRILIAAEKLKKVLSANTTAPFSVESVMDDIDVSSQ 300

301 LSREELEELVKPLLERVTEPVTKALAQAKLSAEEVDFVEIIGGTTRIPTL 350
    ||||||||||:|||:|||.|:|.|||||||:..::|||||||||||||.|
301 LSREELEELVEPLLKRVTYPITNALAQAKLTVNDIDFVEIIGGTTRIPVL 350

351 KQSISEAFGKPLSTTLNQDEAIAKGAAFICAIHSPTLRVRPFKFEDIHPY 400
    |:|||:.||||||:|||||||:|||||||||||||||||||||||||.||
351 KKSISDVFGKPLSSTLNQDEAVAKGAAFICAIHSPTLRVRPFKFEDIDPY 400

401 SVSYSWDKQVEDEDHMEVFPAGSSFPSTKLITLNRTGDFSMAASYTDITQ 450
    ||||:|||||:|||.:|||||.||:||||||||:|||||||.|.||..::
401 SVSYTWDKQVDDEDRLEVFPANSSYPSTKLITLHRTGDFSMKAVYTHPSK 450

451 LPPNTPEQIANWEITGVQLPEGQDSVPVKLKLRCDPSGLHTIEEAYTIED 500
    ||..|...||.|..|||::|:.||.:|||:||||||||||.||.|||.||
451 LPKGTSTTIAKWSFTGVKVPKDQDFIPVKVKLRCDPSGLHIIENAYTTED 500

501 IEVEEPIPLPEDAPEDAEQEFKKVTKTVKKDDLTIVAHTFGLDAKKLNEL 550
    |.|:||:|||||||||||.:||:||||:|||.|.:.|.||.|:..:||:|
501 ITVQEPVPLPEDAPEDAEPQFKEVTKTIKKDVLGMTAKTFALNPVELNDL 550

551 IEKENEMLAQDKLVAETEDRKNTLEEYIYTLRGKLEEEYAPFASDAEKTK 600
    ||||||:..|||||||||||||.|||||||||.||::||:.|||||||.|
551 IEKENELRNQDKLVAETEDRKNALEEYIYTLRAKLDDEYSDFASDAEKEK 600

601 LQGMLNKAEEWLYDEGFDSIKAKYIAKYEELASLGNIIRGRYLAKEEEKK 650
    |:.||...|.|||.:|.||.|||||||||||||||||||||||||||||:
601 LKNMLATTENWLYGDGDDSTKAKYIAKYEELASLGNIIRGRYLAKEEEKR 650

651 QAIRSKQEASQMAAMAEKLAAQRKAEAEKKEEKKDTEGDVDMD        693
    ||:|:.||.|:|..:|||||.||:|.|...:...:.:.::|:|
651 QALRANQETSKMNDIAEKLAEQRRARAASDDSDDNNDENMDLD        693
```

Figure S1 Sse1p (top sequence) and Sse2p alignment. Proteins are 76% identical and 87% similar. Bolded residues are Sse1 mutated residues that alter [*PSI*$^+$] prion propagation. Sse1 residue E504 is in red as this is the only mutated site that is <u>not</u> conserved in Sse2.

```
1    MSTPFGLDLGNNNSVLAVARNRGIDIVVNEVSNRSTPSVVGFGPKNRYLG  50
     ||. .|||:|:.:..:||||..||:.:.||.|:|.||||:.||.|||.:|
1    MSV-VGLDVGSQSCYIAVARAGGIETIANEFSDRCTPSVISFGSKNRTIG  49

51   ETGKNKQTSNIKNTVANLKRIIGLDYHHPDFEQESKHFTSKLVELDDKKT 100
     ...||:|.::..|||:|.||..|..::.|..::|.::.::.||.|.:...
50   VAAKNQQITHANNTVSNFKRFHGRAFNDPFIQKEKENLSYDLVPLKNGGV  99

101  GAEVRFAGEKHVFSATQLAAMFIDKVKDTVKQDTKANITDVCIAVPPWYT 150
     |.:|.:.||:|:||..|:.||.:.|:|:|.:...|..:||..|:||.::|
100  GIKVMYMGEEHLFSVEQITAMLLTKLKETAENSLKKPVTDCVISVPSFFT 149

151  EEQRYNIADAARIAGLNPVRIVNDVTAAGVSYGIFKTDLPEGEEKPRIVA 200
     :.:|.::.|||:|.|||.:|::||:||..::|||:|.|||..:||||||.
150  DAERRSVLDAAQIVGLNCLRLMNDMTAVALNYGIYKQDLPSLDEKPRIVV 199

201  FVDIGHSSYTCSIMAFKKGQLKVLGTACDKHFGGRDFDLAITEHFADEFK 250
     |||:|||::..|..||.||:|||||||.|...||::||..:.|||..|||
200  FVDMGHSAFQVSACAFNKGKLKVLGTAFDPFLGGKNFDEKLVEHFCAEFK 249

251  TKYKIDIRENPKAYNRILTAAEKLKKVLSAN-TNAPFSVESVMNDVDVSS 299
     ||||:|.:...:|..|:....|||||::|:| |:.|.::|..|||.|||.
250  TKYKLDAKSKIRALLRLYQECEKLKKLMSSNSTDLPLNIECFMNDKDVSG 299

300  QLSREELEELVKPLLERVTEPVTKALAQAKLSAEEVDFVEIIGGTTRIPT 349
     :::|.:.|||...||:::..|:...|.|..|..|:|..|||:||.||||.
300  KMNRSQFEELCAELLQKIEVPLYSLLEQTHLKVEDVSAVEIVGGATRIPA 349

350  LKQSISEAFGKPLSTTLNQDEAIAKGAAFICAIHSPTLRVRPFKFEDIHP 399
     :|:.|::.|||.:|||||.|||:|:|.|..|||.||..:||.|...|..|
350  VKERIAKFFGKDISTTLNADEAVARGCALQCAILSPAFKVREFSVTDAVP 399

400  YSVSYSWDKQVEDEDHM-EVFPAGSSFPSTKLITLNRTGDFSMAASYTDI 448
     :.:|..|:...||.:.: |||....:.|.:|::|..|.|.|.:.|.|:|.
400  FPISLIWNHDSEDTEGVHEVFSRNHAAPFSKVLTFLRRGPFELEAFYSDP 449

449  TQLPPNTPE-QIANWEITGVQLPEGQDSVPVKLKLRCDPSGLHTIEEAYT 497
     ..:|  .|| :|..:.:..|...:..:...||:|:|.:..|:.||..|..
450  QGVP--YPEAKIGRFVVQNVSAQKDGEKSRVKVKVRVNTHGIFTISTASM 497

498  IEDIEVEE------------------------------------------ 505
     :|.:..||
498  VEKVPTEENEMSSEADMECLNQRPPENPDTDKNVQQDNSEAGTQPQVQTD 547

506  -------PIPLPE---------DAPE------DAEQEFKKVTKTVKKDDL 533
            | |.||         ||.:      |...|.||....|...:|
548  AQQTSQSP-PSPELTSEENKIPDADKANEKKVDQPPEAKKPKIKVVNVEL 596

534  TIVAH-TFGLDAKKLNELIEKENEMLAQDKLVAETEDRKNTLEEYIYTLR 582
     .|.|: .:.|....||..||.|.:|:.||||..|..|.||.:|||:|..|
597  PIEANLVWQLGKDLLNMYIETEGKMIMQDKLEKERNDAKNAVEEYVYEFR 646

583  GKLEEEYAPFASDAEKTKLQGMLNKAEEWLYDEGFDSIKAKYIAKYEELA 632
     .||...|..|..::.......:|.:.|:|||:||.|..|..|:.|.|||.
647  DKLCGPYEKFICEQDHQNFLRLLTETEDWLYEEGEDQAKQAYVDKLEELM 696

633  SLGNIIRGRYLAKEEEKKQAIRSKQEASQMAAMAEKLAAQRKAEAEK--- 679
     .:|..::.|:...||..|..    :|..|......|:||..:.:.||
697  KIGTPVKVRFQEAEERPKMF----EELGQRLQHYAKIAADFRNKDEKYNH 742

680  --KEEKKDTEGDVD--MD-------------------------------- 693
```

```
        :.|.|..|..|:   |:
743 IDESEMKKVEKSVNEVMEWMNNVMNAQAKKSLDQDPVVRAQEIKTKIKEL 792

693 -------------------------------------------------- 693

793 NNTCEPVVTQPKPKIESPKLERTPNGPNIDKKEEDLEDKNNFGAEPPHQN 842

693 ---------------- 693

843 GECYPNEKNSVNMDLD 858
```

Figure S2 Alignment of Sse1p with HSPH1. Sse1p is top sequence. Proteins are approximately 33% identical and 48% similar. Most highly conserved regions are in the ATPase domain. Nine out of the thirteen Sse1p residues identified as being important in prion propagation are conserved (residues in bold).

Figure S3A

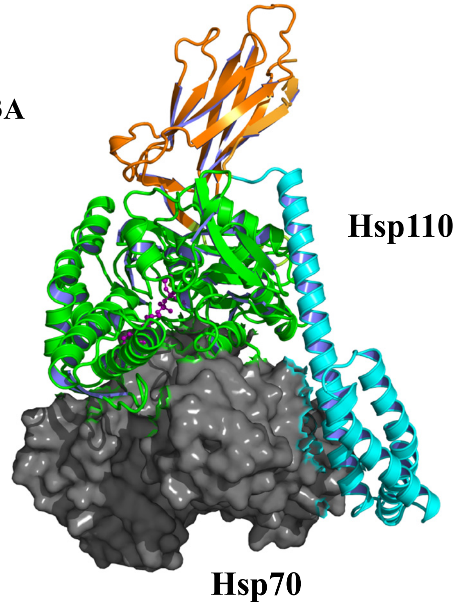

Figure S3B

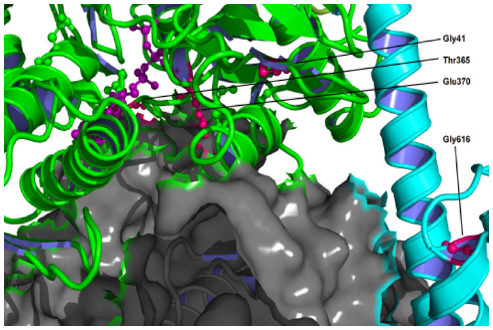

Figure S3 Interaction of Hsp110 with Hsp70. S3(A) Overall structure of Hsp110 in complex with Hsp70. S3(B) Zoomed image showing region of interface between Hsp110 and Hsp70 with highlighted mutated residues that are predicted to influence this interaction.

File S1

Possible Sse1 mutant effects in more detail

Mutants Located in the NBD

P37L (both non-polar hydrophobic residues) – the residue is located in a β strand. Given the proximity to the ATP binding site (within 8Å) – this region could be affected. For example, Asn12, which is in the environment of P37L is predicted to directly hydrogen bond (HB) with ATP so alterations in this region may affect the ATP binding site.

G41D & G50D polar uncharged to polar acidic residues - these mutations could affect the β strand packing as the side chain groups lining one surface of a β-sheet frequently exhibit uniform chemical properties. This region is at the interface between the helical rich extension domain and the NBD and helps to ensure the correct positioning of the former for complex formation with Hsp70. The core of this extensive interface is formed by buried polar interactions of residue Arg47 (of this β strand) with Glu554 and Asp561. Both mutations might induce a local effect on Arg47 and may influence domain-domain interactions.

C211Y (both polar uncharged residues) - Tyr211 is in a β strand and lies in a largely hydrophobic pocket including, Phe201, Val202, Ile213, Pro320, Val321, Ala324 and Phe358 of the NBD. Tyr211 and Cys211 both HB with Ala227 (this interaction is also formed in the Hsp70:Hsp110 complex). This residue is not solvent exposed and its potential role is currently unclear.

D236N (polar acidic to polar uncharged residue) - Asp236 is in an α-helix and forms predicted water mediated interactions with Arg235 and Ser207 (which interacts with ATP). In the Hsp70:Hsp110 complex, Asp236 additionally forms water mediated HBs to Lys86 and His87 as well as a HB to Gly233.

G342D & G343D (polar uncharged to polar acidic residues) – lie in the ATP binding pocket and may affect this interaction.

G342D – G342 HBs with Thr345 and Gln368; D342 additionally forms a water mediated to Mg.

G343D – G343 interacts with ATP but does not form other HBs.
D343 is predicted to HB with Thr344 and Arg346 (which contacts Hsp70 in the complex) and has a predicted water mediated interaction with ATP.

T365I (polar uncharged to non-polar hydrophobic residue)
This residue is in a loop region, which is solvent exposed in the Sse1 un-complexed crystal structure (PDB: 2QXL) and involved in protein-protein contacts with Hsp70 in the 3D2E crystal structure. Thr365 and the modelled Ile365, both HB with Glu339. The side chain conformation may help shape the surface of Sse1p into a cradle for Tyr134 of Hsp70.

E370K (mutating a polar acidic to a polar basic residue)
Glu370 HBs with Arg34 and Asn367 in Sse1 while Lys370 is predicted to form a water mediated interaction with Asn13 (which itself HBs to ATP) and HBs with Asp369, Asn367 (which interacts with Hsp70 in the complex crystal structure) and Lys374 of Sse1. Hence, this mutant could affect the ATP binding site and the Hsp70 interface.

Mutants Located in the SBD

S440L (mutating a polar uncharged to a non-polar hydrophobic residue.)
The side chain is solvent exposed and Leu440 is predicted to HB with Asp407 while there are no HB interactions for Ser440. This mutation may affect substrate binding.

E504K (mutating a polar acidic residue to a polar basic residue)
This residue was not elucidated in the crystal structure and hence was modelled. The modelled Glu504 is solvent exposed, in a loop and is predicted to HB with Lys526 and Glu505. Similarly, Lys504 is predicted to HB with Lys526. Its potential role is currently unclear.

E554K (mutating a polar acidic residue to a polar basic residue)
This residue is located in an α-helix; Glu554 HBs with Leu558 and Arg47; Lys554 HBs with Ile551, Leu558, Leu550 but loses the interaction with Arg47. Glu544 in the helical rich domain is part of the buried polar interactions of the NBD residue Arg47 and may influence domain-domain interactions.

G616D (polar uncharged to polar acidic)
Gly616 is in a loop region in the un-complexed Sse1 structure (PDB: 2QXL) and a helical region in the complexed Sse1 structure (PDB: 3D2E) and forms HBs with Tyr624 and Trp611. It lies in an environment surrounded by Leu612, Asp614, Asp618 and Lys571. Asp616 is modelled in a short α helical region and is predicted to HB with Lys571 and lies near Leu574, Asp614, Leu612 and Trp611. Hence, the residue lies close to the Hsp70 interface and might affect the interaction between the two.
