# Supplementary material for: Mutational Analysis of Sse1 (Hsp110) Suggests an Integral Role for this Chaperone in Yeast Prion Propagation In Vivo
Source: G3 (Bethesda). 2013 Aug 1;3(8):1409–18. doi: 10.1534/g3.113.007112 (PMC3737180; doi:10.1534/g3.113.007112)
Supplement: Supporting Information [file supp_g3.113.007112_FigureS2.pdf]

```
1    MSTPFGLDLGNNNSVLAVARNRGIDIVVNEVSNRSTPSVVGFGPKNRYLG  50
     ||. .|||:|:.:..:||||..||:.:.||.|:|.||||:.||.|||.:|
1    MSV-VGLDVGSQSCYIAVARAGGIETIANEFSDRCTPSVISFGSKNRTIG  49

51   ETGKNKQTSNIKNTVANLKRIIGLDYHHPDFEQESKHFTSKLVELDDKKT 100
     ...||:|.::..|||:|.||..|..::.|..::|.::.:..||.|.:...
50   VAAKNQQITHANNTVSNFKRFHGRAFNDPFIQKEKENLSYDLVPLKNGGV  99

101  GAEVRFAGEKHVFSATQLAAMFIDKVKDTVKQDTKANITDVCIAVPPWYT 150
     |.:|.:.||:|:||..|:.||.:.|:|:|.:...|..:||..|:||.::|
100  GIKVMYMGEEHLFSVEQITAMLLTKLKETAENSLKKPVTDCVISVPSFFT 149

151  EEQRYNIADAARIAGLNPVRIVNDVTAAGVSYGIFKTDLPEGEEKPRIVA 200
     :.:|.::.|||:|.|||.:|::||:||..::|||:|.|||..:||||||.
150  DAERRSVLDAAQIVGLNCLRLMNDMTAVALNYGIYKQDLPSLDEKPRIVV 199

201  FVDIGHSSYTCSIMAFKKGQLKVLGTACDKHFGGRDFDLAITEHFADEFK 250
     |||:|||::..|..||.||:|||||||.|...||::||..:.|||..|||
200  FVDMGHSAFQVSACAFNKGKLKVLGTAFDPFLGGKNFDEKLVEHFCAEFK 249

251  TKYKIDIRENPKAYNRILTAAEKLKKVLSAN-TNAPFSVESVMNDVDVSS 299
     ||||:|.:...:|..|:....|||||::|:| |:.|.::|..|||.|||.
250  TKYKLDAKSKIRALLRLYQECEKLKKLMSSNSTDLPLNIECFMNDKDVSG 299

300  QLSREELEELVKPLLERVTEPVTKALAQAKLSAEEVDFVEIIGGTTRIPT 349
     :::|.:.|||...||:::..|:...|.|..|..|:|..|||:||.||||.
300  KMNRSQFEELCAELLQKIEVPLYSLLEQTHLKVEDVSAVEIVGGATRIPA 349

350  LKQSISEAFGKPLSTTLNQDEAIAKGAAFICAIHSPTLRVRPFKFEDIHP 399
     :|:.|::.|||.:|||||.|||:|:|.|..|||.||..:||.|...|..|
350  VKERIAKFFGKDISTTLNADEAVARGCALQCAILSPAFKVREFSVTDAVP 399

400  YSVSYSWDKQVEDEDHM-EVFPAGSSFPSTKLITLNRTGDFSMAASYTDI 448
     :.:|..|:...||.:.: |||....:.|.:|::|..|.|.|.:.|.|:|.
400  FPISLIWNHDSEDTEGVHEVFSRNHAAPFSKVLTFLRRGPFELEAFYSDP 449

449  TQLPPNTPE-QIANWEITGVQLPEGQDSVPVKLKLRCDPSGLHTIEEAYT 497
     ..:|  .|| :|..:.:..|...:..:...||:|:|.:..|:.||..|..
450  QGVP--YPEAKIGRFVVQNVSAQKDGEKSRVKVKVRVNTHGIFTISTASM 497

498  IEDIEVEE------------------------------------------ 505
     :|.:..||
498  VEKVPTEENEMSSEADMECLNQRPPENPDTDKNVQQDNSEAGTQPQVQTD 547

506  -------PIPLPE---------DAPE------DAEQEFKKVTKTVKKDDL 533
            | |.||         ||.:      |...|.||....|...:|
548  AQQTSQSP-PSPELTSEENKIPDADKANEKKVDQPPEAKKPKIKVVNVEL 596

534  TIVAH-TFGLDAKKLNELIEKENEMLAQDKLVAETEDRKNTLEEYIYTLR 582
     .|.|: .:.|....||..||.|.:|:.||||..|..|.||.:|||:|..|
597  PIEANLVWQLGKDLLNMYIETEGKMIMQDKLEKERNDAKNAVEEYVYEFR 646

583  GKLEEEYAPFASDAEKTKLQGMLNKAEEWLYDEGFDSIKAKYIAKYEELA 632
     .||...|..|..:.:......:|.:.|:|||:||.|..|..|:.|.|||.
647  DKLCGPYEKFICEQDHQNFLRLLTETEDWLYEEGEDQAKQAYVDKLEELM 696

633  SLGNIIRGRYLAKEEEKKQAIRSKQEASQMAAMAEKLAAQRKAEAEK--- 679
     .:|..::.|:...||..|..    :|..|......|:||..:.:.||
697  KIGTPVKVRFQEAEERPKMF----EELGQRLQHYAKIAADFRNKDEKYNH 742

680  --KEEKKDTEGDVD--MD-------------------------------- 693
```

```
      :.|.|..|..|:   |:
743 IDESEMKKVEKSVNEVMEWMNNVMNAQAKKSLDQDPVVRAQEIKTKIKEL 792

693 -------------------------------------------------- 693

793 NNTCEPVVTQPKPKIESPKLERTPNGPNIDKKEEDLEDKNNFGAEPPHQN 842

693 ---------------- 693

843 GECYPNEKNSVNMDLD 858
```

Figure S2 Alignment of Sse1p with HSPH1. Sse1p is top sequence. Proteins are approximately 33% identical and 48% similar. Most highly conserved regions are in the ATPase domain. Nine out of the thirteen Sse1p residues identified as being important in prion propagation are conserved (residues in bold).
