# Supplementary material for: Mutational Analysis of Sse1 (Hsp110) Suggests an Integral Role for this Chaperone in Yeast Prion Propagation In Vivo
Source: G3 (Bethesda). 2013 Aug 1;3(8):1409–18. doi: 10.1534/g3.113.007112 (PMC3737180; doi:10.1534/g3.113.007112)
Supplement: Supporting Information [file supp_g3.113.007112_FigureS3.pdf]

Figure S3A

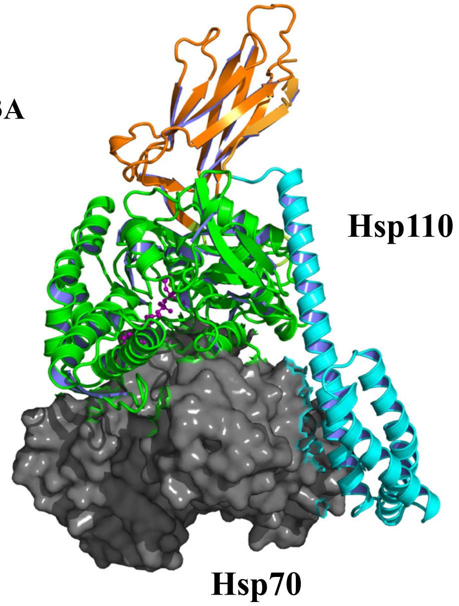

Figure S3B

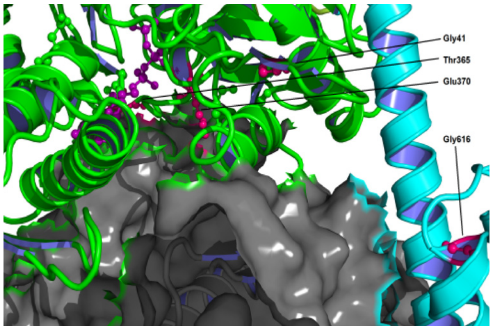

Figure S3 Interaction of Hsp110 with Hsp70. S3(A) Overall structure of Hsp110 in complex with Hsp70. S3(B) Zoomed image showing region of interface between Hsp110 and Hsp70 with highlighted mutated residues that are predicted to influence this interaction.
