# Supplementary material for: Mutational Analysis of Sse1 (Hsp110) Suggests an Integral Role for this Chaperone in Yeast Prion Propagation In Vivo
Source: G3 (Bethesda). 2013 Aug 1;3(8):1409–18. doi: 10.1534/g3.113.007112 (PMC3737180; doi:10.1534/g3.113.007112)
Supplement: Supporting Information [file supp_g3.113.007112_FileS1.pdf]

File S1

Possible Sse1 mutant effects in more detail

Mutants Located in the NBD

P37L (both non-polar hydrophobic residues) – the residue is located in a β strand. Given the proximity to the ATP binding site (within 8Å) – this region could be affected. For example, Asn12, which is in the environment of P37L is predicted to directly hydrogen bond (HB) with ATP so alterations in this region may affect the ATP binding site.

G41D & G50D polar uncharged to polar acidic residues - these mutations could affect the β strand packing as the side chain groups lining one surface of a β-sheet frequently exhibit uniform chemical properties. This region is at the interface between the helical rich extension domain and the NBD and helps to ensure the correct positioning of the former for complex formation with Hsp70. The core of this extensive interface is formed by buried polar interactions of residue Arg47 (of this β strand) with Glu554 and Asp561. Both mutations might induce a local effect on Arg47 and may influence domain-domain interactions.

C211Y (both polar uncharged residues) - Tyr211 is in a β strand and lies in a largely hydrophobic pocket including, Phe201, Val202, Ile213, Pro320, Val321, Ala324 and Phe358 of the NBD. Tyr211 and Cys211 both HB with Ala227 (this interaction is also formed in the Hsp70:Hsp110 complex). This residue is not solvent exposed and its potential role is currently unclear.

D236N (polar acidic to polar uncharged residue) - Asp236 is in an α-helix and forms predicted water mediated interactions with Arg235 and Ser207 (which interacts with ATP). In the Hsp70:Hsp110 complex, Asp236 additionally forms water mediated HBs to Lys86 and His87 as well as a HB to Gly233.

G342D & G343D (polar uncharged to polar acidic residues) – lie in the ATP binding pocket and may affect this interaction.

G342D – G342 HBs with Thr345 and Gln368; D342 additionally forms a water mediated to Mg.

G343D – G343 interacts with ATP but does not form other HBs.
D343 is predicted to HB with Thr344 and Arg346 (which contacts Hsp70 in the complex) and has a predicted water mediated interaction with ATP.

T365I (polar uncharged to non-polar hydrophobic residue)
This residue is in a loop region, which is solvent exposed in the Sse1 un-complexed crystal structure (PDB: 2QXL) and involved in protein-protein contacts with Hsp70 in the 3D2E crystal structure. Thr365 and the modelled Ile365, both HB with Glu339. The side chain conformation may help shape the surface of Sse1p into a cradle for Tyr134 of Hsp70.

E370K (mutating a polar acidic to a polar basic residue)
Glu370 HBs with Arg34 and Asn367 in Sse1 while Lys370 is predicted to form a water mediated interaction with Asn13 (which itself HBs to ATP) and HBs with Asp369, Asn367 (which interacts with Hsp70 in the complex crystal structure) and Lys374 of Sse1. Hence, this mutant could affect the ATP binding site and the Hsp70 interface.

Mutants Located in the SBD

S440L (mutating a polar uncharged to a non-polar hydrophobic residue.)
The side chain is solvent exposed and Leu440 is predicted to HB with Asp407 while there are no HB interactions for Ser440. This mutation may affect substrate binding.

E504K (mutating a polar acidic residue to a polar basic residue)
This residue was not elucidated in the crystal structure and hence was modelled. The modelled Glu504 is solvent exposed, in a loop and is predicted to HB with Lys526 and Glu505. Similarly, Lys504 is predicted to HB with Lys526. Its potential role is currently unclear.

E554K (mutating a polar acidic residue to a polar basic residue)
This residue is located in an α-helix; Glu554 HBs with Leu558 and Arg47; Lys554 HBs with Ile551, Leu558, Leu550 but loses the interaction with Arg47. Glu544 in the helical rich domain is part of the buried polar interactions of the NBD residue Arg47 and may influence domain-domain interactions.

G616D (polar uncharged to polar acidic)
Gly616 is in a loop region in the un-complexed Sse1 structure (PDB: 2QXL) and a helical region in the complexed Sse1 structure (PDB: 3D2E) and forms HBs with Tyr624 and Trp611. It lies in an environment surrounded by Leu612, Asp614, Asp618 and Lys571. Asp616 is modelled in a short α helical region and is predicted to HB with Lys571 and lies near Leu574, Asp614, Leu612 and Trp611. Hence, the residue lies close to the Hsp70 interface and might affect the interaction between the two.
