# Supplementary material for: Mutational Analysis of Sse1 (Hsp110) Suggests an Integral Role for this Chaperone in Yeast Prion Propagation In Vivo
Source: G3 (Bethesda). 2013 Aug 1;3(8):1409–18. doi: 10.1534/g3.113.007112 (PMC3737180; doi:10.1534/g3.113.007112)
Supplement: Supporting Information [file supp_g3.113.007112_FigureS1.pdf]

```
  1 MSTPFGLDLGNNNSVLAVARNRGIDIVVNEVSNRSTPSVVGFGPKNRYLG 50
    |||||||||||||||||||||||||:||||||||||||:|||||:|||||
  1 MSTPFGLDLGNNNSVLAVARNRGIDVVVNEVSNRSTPSLVGFGPRNRYLG 50

 51 ETGKNKQTSNIKNTVANLKRIIGLDYHHPDFEQESKHFTSKLVELDDKKT 100
    |:||.|||||:||||.||||||||.:..|:|:.|:|.||||||:|.:.|.
 51 ESGKTKQTSNVKNTVENLKRIIGLKFKDPEFDIENKFFTSKLVQLKNGKV 100

101 GAEVRFAGEKHVFSATQLAAMFIDKVKDTVKQDTKANITDVCIAVPPWYT 150
    |.||.|.|:.||||||||.||||||||.||:::||::|||||:|||.||:
101 GVEVEFGGKTHVFSATQLTAMFIDKVKHTVQEETKSSITDVCLAVPVWYS 150

151 EEQRYNIADAARIAGLNPVRIVNDVTAAGVSYGIFKTDLPEGEEKPRIVA 200
    ||||||||||||||||||||||||||||.||||:||.|||..||||||:.
151 EEQRYNIADAARIAGLNPVRIVNDVTAAAVSYGVFKNDLPGPEEKPRIIG 200

201 FVDIGHSSYTCSIMAFKKGQLKVLGTACDKHFGGRDFDLAITEHFADEFK 250
    .||||||:||||||||:||::||||||.||||||||||.||||||||:||
201 LVDIGHSTYTCSIMAFRKGEMKVLGTAYDKHFGGRDFDRAITEHFADQFK 250

251 TKYKIDIRENPKAYNRILTAAEKLKKVLSANTNAPFSVESVMNDVDVSSQ 300
    .|||||||:|||||||||.|||||||||||||.|||||||||:|:|||||
251 DKYKIDIRKNPKAYNRILIAAEKLKKVLSANTTAPFSVESVMDDIDVSSQ 300

301 LSREELEELVKPLLERVTEPVTKALAQAKLSAEEVDFVEIIGGTTRIPTL 350
    ||||||||||:|||:|||.|:|.|||||||:..::|||||||||||||.|
301 LSREELEELVEPLLKRVTYPITNALAQAKLTVNDIDFVEIIGGTTRIPVL 350

351 KQSISEAFGKPLSTTLNQDEAIAKGAAFICAIHSPTLRVRPFKFEDIHPY 400
    |:|||:.||||||:|||||||:|||||||||||||||||||||||||.||
351 KKSISDVFGKPLSSTLNQDEAVAKGAAFICAIHSPTLRVRPFKFEDIDPY 400

401 SVSYSWDKQVEDEDHMEVFPAGSSFPSTKLITLNRTGDFSMAASYTDITQ 450
    ||||:|||||:|||.:|||||.||:||||||||:|||||||.|.||..::
401 SVSYTWDKQVDDEDRLEVFPANSSYPSTKLITLHRTGDFSMKAVYTHPSK 450

451 LPPNTPEQIANWEITGVQLPEGQDSVPVKLKLRCDPSGLHTIEEAYTIED 500
    ||..|...||.|..|||::|:.||.:|||:||||||||||.||.|||.||
451 LPKGTSTTIAKWSFTGVKVPKDQDFIPVKVKLRCDPSGLHIIENAYTTED 500

501 IEVEEPIPLPEDAPEDAEQEFKKVTKTVKKDDLTIVAHTFGLDAKKLNEL 550
    |.|:||:|||||||||||.:||:||||:|||.|.:.|.||.|:..:||:|
501 ITVQEPVPLPEDAPEDAEPQFKEVTKTIKKDVLGMTAKTFALNPVELNDL 550

551 IEKENEMLAQDKLVAETEDRKNTLEEYIYTLRGKLEEEYAPFASDAEKTK 600
    ||||||:..|||||||||||||.|||||||||.||::||:.|||||||.|
551 IEKENELRNQDKLVAETEDRKNALEEYIYTLRAKLDDEYSDFASDAEKEK 600

601 LQGMLNKAEEWLYDEGFDSIKAKYIAKYEELASLGNIIRGRYLAKEEEKK 650
    |:.||...|.|||.:|.||.|||||||||||||||||||||||||||||:
601 LKNMLATTENWLYGDGDDSTKAKYIAKYEELASLGNIIRGRYLAKEEEKR 650

651 QAIRSKQEASQMAAMAEKLAAQRKAEAEKKEEKKDTEGDVDMD        693
    ||:|:.||.|:|..:|||||.||:|.|...:...:.:.::|:|
651 QALRANQETSKMNDIAEKLAEQRRARAASDDSDDNNDENMDLD        693
```

Figure S1 Sse1p (top sequence) and Sse2p alignment. Proteins are 76% identical and 87% similar. Bolded residues are Sse1 mutated residues that alter [*PSI*$^+$] prion propagation. Sse1 residue E504 is in red as this is the only mutated site that is not conserved in Sse2.
